# Better together: the use of virus-induced gene silencing technique to repress the expression of two endogenous citrus genes simultaneously

**DOI:** 10.1080/15592324.2022.2106079

**Published:** 2022-08-02

**Authors:** Nabil Killiny

**Affiliations:** Department of Plant Pathology, Citrus Research and Education Center, IFAS, University of Florida, Lake Alfred, FL, USA

**Keywords:** Virus-induced gene silencing, Citrus tristeza virus, phytoene desaturase, delta (δ)-aminolevulinic acid dehydratase, functional genomics

## Abstract

Virus-induced gene silencing is a promising technique for functional genomics studies. *Citrus tristeza virus* was employed successfully to create an infectious clone that was used to silence endogenous citrus genes. Phytoene desaturase (*PDS*) and delta (δ)-aminolevulinic acid dehydratase (*ALAD*) were targeted successfully in citrus. Silencing *PDS* usually results in a photo-bleached leaf phenotype while silencing *ALAD* causes discrete yellow spots in leaves. Silencing two or more genes simultaneously using the same infectious clone could be difficult due to the capacity of the plasmid and subsequent cloning. On the other hand, inoculating a new construct into a citrus plant pre-infected with another construct fails due to the superinfection exclusion phenomenon. Herein, I report our successful trials whereby we simultaneously graft-inoculate constructs targeting *PDS* and *ALAD*. The budwoods were graft-inoculated into the same tree but on two different branches. Interestingly, a new phenotype was produced because of the silencing of the two genes, which we called “color-breaking”. The phenotype was observed in both branches. Gene expression analysis showed a significant reduction of *PDS* and *ALAD* transcripts. This finding suggests the possibility of targeting more than one gene using different constructs, however, the graft-inoculation must be at the same time.

## Main text

Virus-induced gene silencing technique (VIGS) has been commonly employed to study the functional genomics of genes in plants.^1^ VIGS has been successfully used in a huge number of plant species including *Arabidopsis thaliana, Nicotiana tabacum, Solanum lycopersicon*, and *Citrus* spp.^2,3^ Plant RNAse III-like enzyme (Dicer-like endonuclease) cleaves viral dsRNAs resulting in small interfering RNAs (siRNAs).^4^ The siRNAs integrate with the RNA-induced silencing complex (RISC), Argonaute, and other associated proteins. The complex recognizes and guides the degradation of the homologous regions in the viral RNA.^5^

The VIGS vectors are created by cloning the entire genome of a plant virus into a backbone plasmid. By integrating a truncated sequence of the host gene, the defense machinery will target the corresponding mRNAs and cause downregulation of the gene expression.^6^ The efficiency of the VIGS technique depends on the movement and the distribution of viral particles within the plants.^1^ Therefore, it is suggested to use an extra reporter gene such as phytoene desaturase (*PDS*) to measure the amount of silencing of the targeted gene.^7^ Silencing the reporter gene usually causes a specific phenotype, photobleaching, in the case of *PDS*.^7^

In citrus, VIGS is accomplished using the mild strain T-36 of *Citrus tristeza virus* (CTV), a member of the genus *Closterovirus*, to knock down endogenous genes. The use of this infectious clone provides many advantages: (i) it does not provoke any symptoms in inoculated citrus; (ii) it can express exogenous genes in citrus and remains for years;^8^ (iii) it can be used in the field to protect citrus from pests or diseases;^9^ and (iv) more than one exogenous gene could be inserted into the infectious clone simultaneously.^8,10^ Previously, we demonstrated that the wild-type CTV-T-36 (CTV-wt) does not cause any dramatic effects on the phloem sap composition and released volatiles of *Citrus macrophylla*.^11^

**Figure 1.**
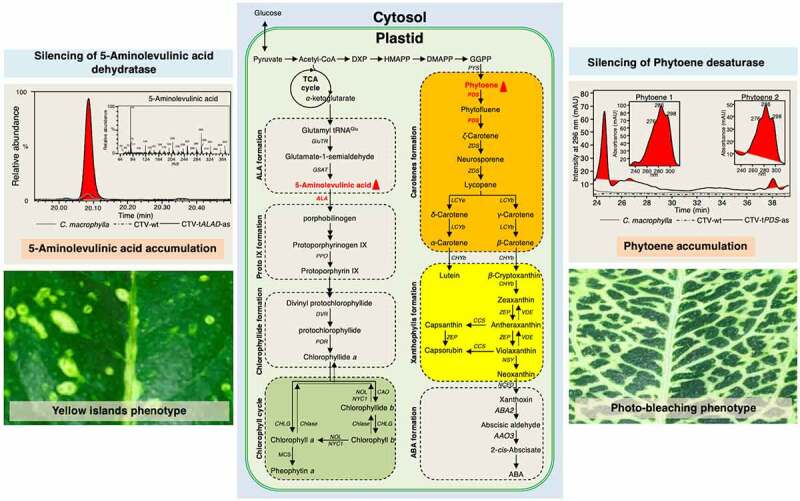
**The effect of silencing phytoene desaturase (*PDS*) and delta (δ)-aminolevulinic acid dehydratase (*ALAD*) on carotenoid and chlorophyll pathways and leaf phenotypes**. Silencing *PDS* causes accumulation of phytoene and reduction in carotenoid production seen as a photo-bleaching phenotype. Silencing *ALAD* causes accumulation of δ-aminolevulinic acid and reduction in chlorophyll production observed as discrete yellow islands phenotype. Chromatograms and schematic representation of carotenoid and chlorophyll pathways inside the plastids were adapted and combined from Killiny et al.^2,3^

Among the genes targeted using CTV is the reporter gene, phytoene desaturase (*PDS*), a key plant enzyme involved in carotenoid biosynthesis.^12^ Silencing the *PDS* gene in citrus causes “photo-bleached leaf” phenotype (Figure 1).^12^ Silencing the citrus *PDS* gene reduces the production of carotenoids and accumulates the precursor phytoene (Figure 1). Furthermore, we showed that the use of the antisense orientation of the construct (CTV-t*PDS*-as) causes a stronger photobleached-leaf phenotype compared to the sense orientation (CTV-t*PDS*).^12^ Although the expression *of PDS* gene was down-regulated when sense and antisense orientations of truncated sequences were used to build the construct, superior silencing of *PDS* and stronger phenotype were achieved with the antisense orientation due to the production of subgenomic RNAs that complemented the small interfering RNAs.^12^

Likewise, we have targeted delta (δ)-aminolevulinic acid dehydratase (ALAD), which is a key enzyme in tetrapyrrole synthesis, and an important intermediate for chlorophyll biosynthesis using CTV.^3^ *Citrus macrophylla* plants inoculated with CTV-t*ALAD*-as virions possess a specific phenotype including discrete yellow spots or “islands” (Figure 1). In addition to the yellow islands, leaf necrosis on stem and apical meristem were observed.^3^ Silencing *ALAD* causes a reduction in chlorophyll production and accumulation of the precursor δ-aminolevulinic acid (Figure 1).

One major challenge in the VIGS technique is targeting more than a single gene at once due to the limited capacity of the infectious clone plasmid to contain long inserts. This fact may lead us to think about using two different constructs each has a single truncated gene sequence. However, the phenomenon of homologous interference, also known as superinfection exclusion, may occur and one of the constructs may be excluded. The phenomenon of superinfection exclusion is well known in viral diseases, wherein the first established viral infection blocks a secondary infection with the same or a closely related virus.^13^

For CTV, the phenomenon has been reported and well-studied.^14^ It was shown that superinfection exclusion happened only between isolates of the same strain. When isolates of the same strain were used for sequential plant inoculation, the primary infection provided perfect exclusion of the second isolate. For example, primary infection with CTV-T36 prevented superinfection with the infectious clone T36-based GFP-expressing CTV (CTV-T36-GFP).^14^ This phenomenon has been well employed for the cross protection as a strategy to prevent the second infection in the field.^14^

**Figure 2.**
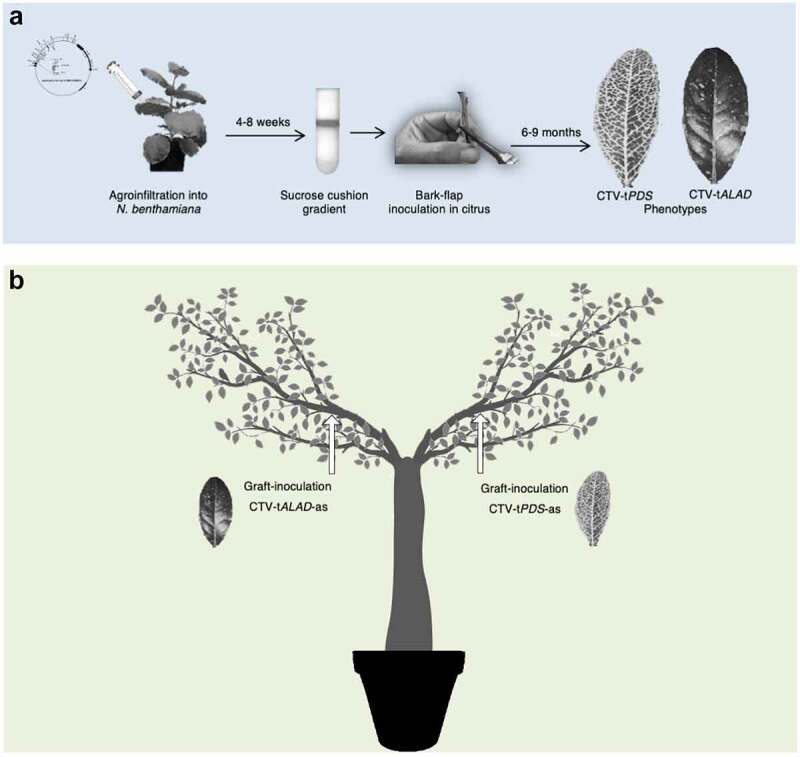
**Experimental procedures used in the study**. (a) Process of CTV-based infectious clone propagation in *N. benthamiana* and inoculation into *C. macrophylla*. (b) Y-shaped trees received CTV-t*PDS*-as and CTV-t*ALAD*-as budwood grafts into separate branches.

Herein, we report our trials to infect *C. macrophylla* with both CTV-t*ALAD*-as and CTV-t*PDS*-as to test if we could repress two genes using two different constructs of the CTV-T-36 based infectious clone. The source trees infected with CTV vectors were produced in our previous work.^2,3^ Briefly, the CTV constructs were agroinfiltrated into *Nicotiana benthamiana* followed by virion purification using a sucrose cushion gradient, and purified virions were bark-flap inoculated into *C. macrophylla* (Figure 2a).

**Figure 3.**
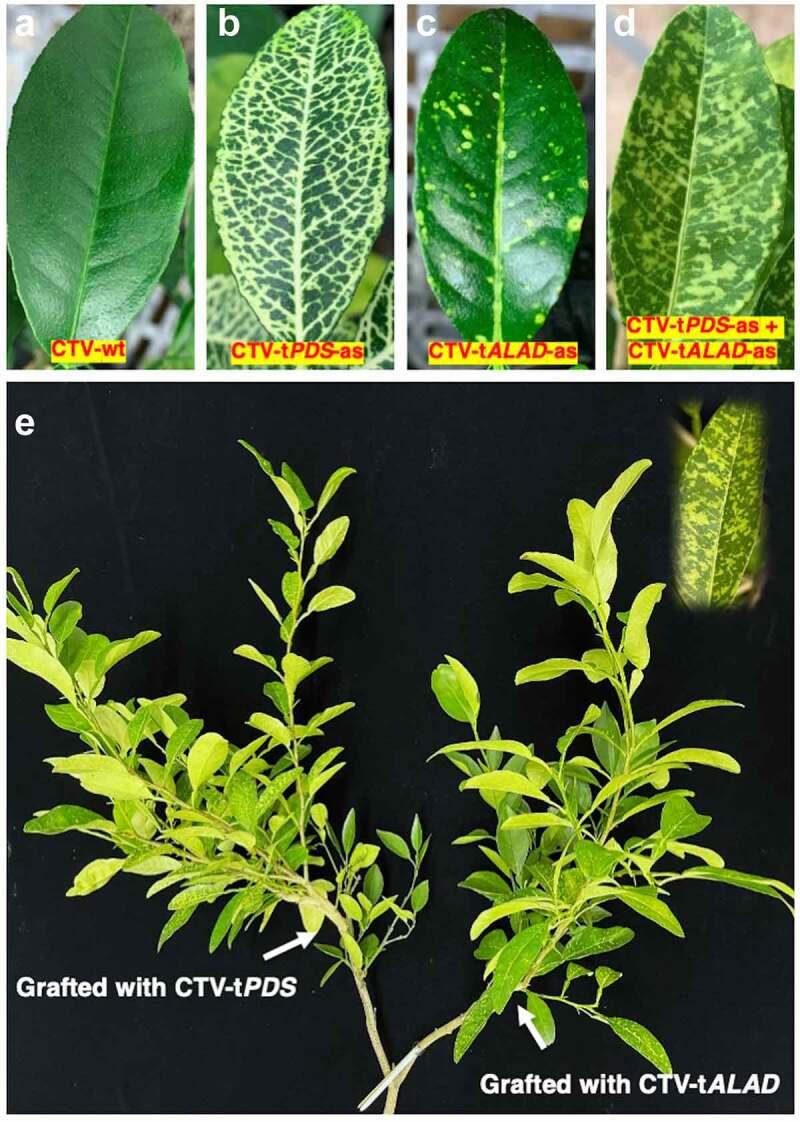
**Leaf phenotypes as a result of silencing *PDS* and/or *ALAD.*** (a) Control leaf (from trees infected with CTV-wt). (b) Photo-bleaching phenotype (from trees infected with CTV-t*PDS*-as). (c) Yellow islands phenotype (from trees infected with CTV-t*ALAD*-as). (d) Color-breaking phenotype (from trees inoculated simultaneously with CTV-t*PDS*-as and CTV-t*ALAD*-as into two different branches). (e) The Y-shaped tree inoculated simultaneously with CTV-t*PDS*-as and CTV-t*ALAD*-as into two different branches.

In the first trials, we grafted budwood from *C. macrophylla* infected with one construct onto *C. macrophylla* plants pre-infected with the other construct and possessing its specific phenotype (either budwood infected with CTV-t*PDS*-as onto infected *C. macrophylla* with CTV-t*ALAD*-as or *vice-versa*). In these trees, we did not observe the newly grafted phenotype, only the original phenotype for each group. Therefore, we decided to perform another trial grafting both constructs onto the same healthy plant simultaneously. We created “Y-shaped” trees by selectively trimming branches to form two distinct main branches. On these trees, both branches were side-grafted, one branch with budwood harboring the CTV-t*PDS*-as and the other with budwood harboring CTV-t*ALAD*-as (Figure 2b). After approximately one year, a new phenotype was visible in both branches of the Y-shaped trees. The new phenotype was distinctly different from either individual phenotype rather than a combination or overlapping of the two phenotypes. That was expected if both vectors multiply and produce small interfering RNA targeting both genes. We called this new phenotype “color-breaking” due to the irregular chlorosis pattern (Figure 3). The development of the color-breaking phenotype in both branches indicated that either the CTV virions or the small interfering RNAs resulting from the two constructs moved from branch to branch systemically creating the new color-breaking phenotype. This finding suggests that the simultaneous inoculation with two CTV constructs targeting two different genes could prevent superinfection exclusion.

**Figure 4.**
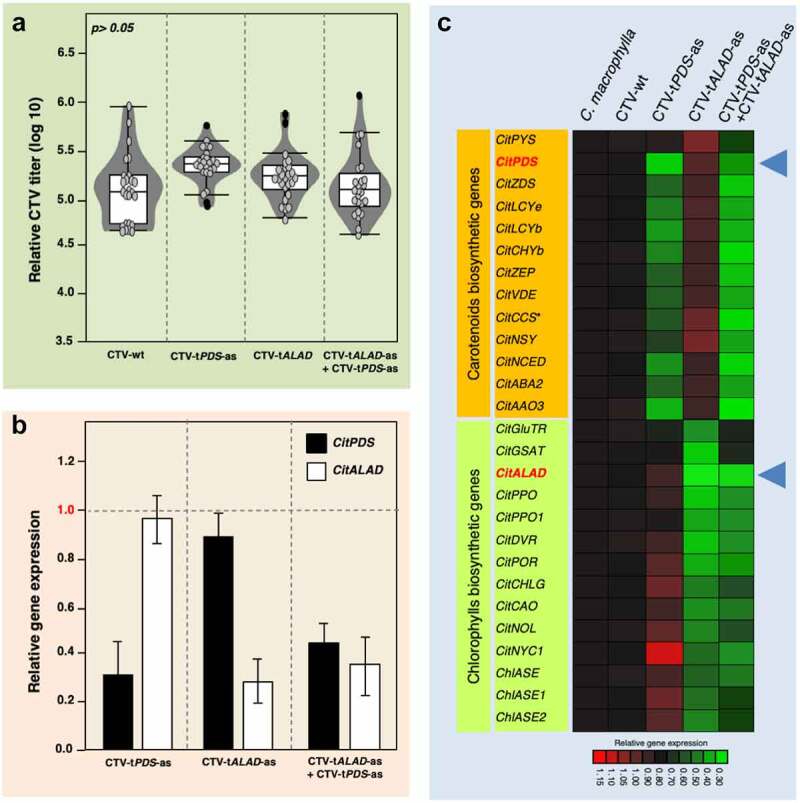
**Virus titers and gene expression profiles in trees graft-inoculated with CTV-t*PDS*-as and/or CTV-t*ALAD*-as**. (a) CTV titers calculated using RT-PCR. (b) Transcripts of *PDS* and *ALAD* in infected trees with CTV-t*PDS*-as and/or CTV-t*ALAD*-as normalized to transcripts in CTV-wt infected trees (control). (c) Heat map for the expression of genes implicated in carotenoid and chlorophyll pathways. Blue arrow heads indicate the silenced genes *PDS* and *ALAD.*

CTV titers were measured using quantitative PCR as described by Harper et al.^15^ The CTV populations were very comparable in all treatments (Figure 4a), suggesting that the CTV population may reach a cap even if it consists of more than one construct. Gene expression for *PDS, ALAD*, and genes implicated in their pathways was carried out as described in previous studies.^2,3^ The expressions of *PDS* and *ALAD* in the double-inoculated plants were reduced confirming the RNAi effect (Figure 4b). In fact, the expression profiles of genes implicated in the carotenoid and chlorophyll biosynthetic pathways were affected in the double-inoculated trees justifying the color-breaking phenotype (Figure 4c).

In summary, this work suggests that simultaneous inoculation with two constructs of the same infectious clone containing two different genes allows repression of the expression of both and causes effective RNA interference. The work provides a simple solution when a virus infectious clone plasmid cannot carry more than one truncated gene, and when the goal of the study is to knockdown two genes. This solution will help in functional genomic studies, especially those exploring the relationships between two biosynthetic pathways.
